# Human Serum Extracellular Vesicle Proteomic Profile Depends on the Enrichment Method Employed

**DOI:** 10.3390/ijms222011144

**Published:** 2021-10-15

**Authors:** Mikel Azkargorta, Ibon Iloro, Iraide Escobes, Diana Cabrera, Juan M. Falcon-Perez, Felix Elortza, Felix Royo

**Affiliations:** 1 Center for Cooperative Research in Biosciences (CIC bioGUNE), Basque Research and Technology Alliance (BRTA), 48160 Derio, Spain; mazkargorta@cicbiogune.es (M.A.); iiloro@cicbiogune.es (I.I.); iescobes@cicbiogune.es (I.E.); dcabrera@cicbiogune.es (D.C.); jfalcon@cicbiogune.es (J.M.F.-P.); 2 Network Center of Cooperative Research in Biomedicine of Hepatic and Digestive Diseases (CIBERehd), 28029 Madrid, Spain; 3 Ikerbasque, Basque Foundation for Science, 48013 Bilbao, Spain

**Keywords:** extracellular vesicles, ultracentrifugation, size exclusion, precipitation kits, proteomic, serum

## Abstract

The proteomic profiling of serum samples supposes a challenge due to the large abundance of a few blood proteins in comparison with other circulating proteins coming from different tissues and cells. Although the sensitivity of protein detection has increased enormously in the last years, specific strategies are still required to enrich less abundant proteins and get rid of abundant proteins such as albumin, lipoproteins, and immunoglobulins. One of the alternatives that has become more promising is to characterize circulating extracellular vesicles from serum samples that have great interest in biomedicine. In the present work, we enriched the extracellular vesicles fraction from human serum by applying different techniques, including ultracentrifugation, size-exclusion chromatography, and two commercial precipitation methods based on different mechanisms of action. To improve the performance and efficacy of the techniques to promote purity of the preparations, we have employed a small volume of serum samples (<100 mL). The comparative proteomic profiling of the enriched preparations shows that ultracentrifugation procedure yielded a larger and completely different set of proteins than other techniques, including mitochondrial and ribosome related proteins. The results showed that size exclusion chromatography carries over lipoprotein associated proteins, while a polymer-based precipitation kit has more affinity for proteins associated with granules of platelets. The precipitation kit that targets glycosylation molecules enriches differentially protein harboring glycosylation sites, including immunoglobulins and proteins of the membrane attack complex.

## 1. Introduction

As a source of biomarkers, blood is one of the most attractive biofluids. Blood-based proteomic biomarkers are potentially inexpensive and practical to implement, allowing for repeated sampling in large cohorts. Therefore, blood biomarkers might have significant advantages over other biomarker modalities. Moreover, the popularization of the concept of liquid biopsy, based on the assessment of circulating molecules in the blood of patient, to extract the molecular information of primary tumor, metabolic diseases, and monitoring disease progression on solid organs [1,2]. 

However, from a proteomic perspective, both serum and plasma represent a very complex and analytically challenging sample. They present a mixture of free and macromolecule-bound proteins with a wide range of concentrations, from mg to ng per mL, where 30 of the most abundant proteins comprise more than 99 percent of its total protein mass. This makes the detection of low abundance serum proteins by untargeted proteomics very difficult [3]. As a way of overcoming this problem, particular care has been appointed to the study of extracellular vesicles (EVs), as a valuable component of the blood. Indeed, EVs are circulating entities that carry precious information about their parental cells. They were reckoned as very valuable source of markers for many types of diseases such cancer [4], Alzheimer’s and other neurodegenerative diseases [5,6], hematological malignancies and coagulopathies [7,8], and several liver diseases [9], as well as parasitic diseases such malaria [10]. It is also important to emphasize that EVs are not only cargo carriers, but contain pro- or antioxidant machinery able to produce reactive oxidative species and therefore can modify the reactive oxidative content in the extracellular compartments [11].

**Figure 1:**
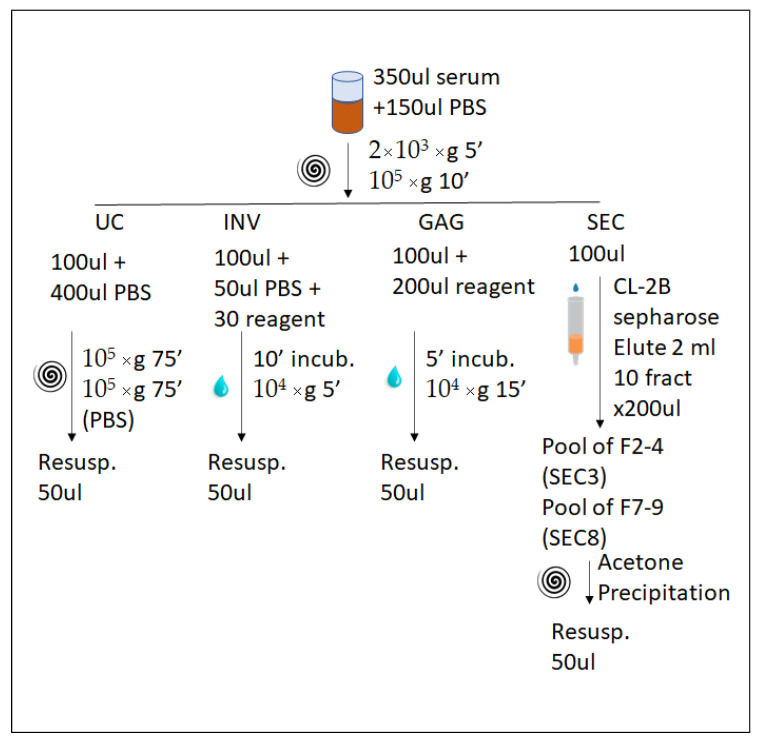
Schematic representation of the EV separation design. Each serum was diluted (the 100 µL loaded for each test contains 80 µL of undiluted serum), cleared and subsequently divided in four aliquots processed by four different techniques, ultracentrifugation (UC), INV (Total Exosome Purification kit, Invitrogen, Thermo Fisher Scientific, Waltham, MA, USA), GAG (Exo-GAG precipitation solution, Nasabiotech, A Coruña, Spain) and SEC (size exclusion chromatography). The results presented in this study were obtained from four independent biological replicates.

For that reason, EVs analysis has become a key element for liquid biopsy protocols applicable to clinical routine [12,13], with the publication of guidelines to try to unify criteria for the researcher in this field [14]. Regarding proteomic analysis, several studies have recently been conducted to optimize the conditions to obtain high purity EVs [15] or compare different methods for EV purification [16]. While those studies start from 1 mL up to several mL of serum and/or plasma, many researchers try to get the most from small volumes of sample, and results may be quite different depending on the technique employed. In the present study, we have compared four different techniques: ultracentrifugation (UC); two commercial kits for precipitation, one based in polymer precipitation (INV) and the other in a molecule with affinity for glycosylated groups (GAG); and the fourth method based on size-exclusion chromatography (SEC). As a starting sample, we have chosen a small volume of serum (~80 µL, initially diluted up to 100 µL with PBS), and subsequently to perform a proteomics analysis (Figure 1). The results obtained showed that ultracentrifugation and the rest of the techniques enrich a different subset of proteins.

## 2. Results

### 2.1. Characterization of Vesicles Separated by Different Techniques

**Figure 2:**
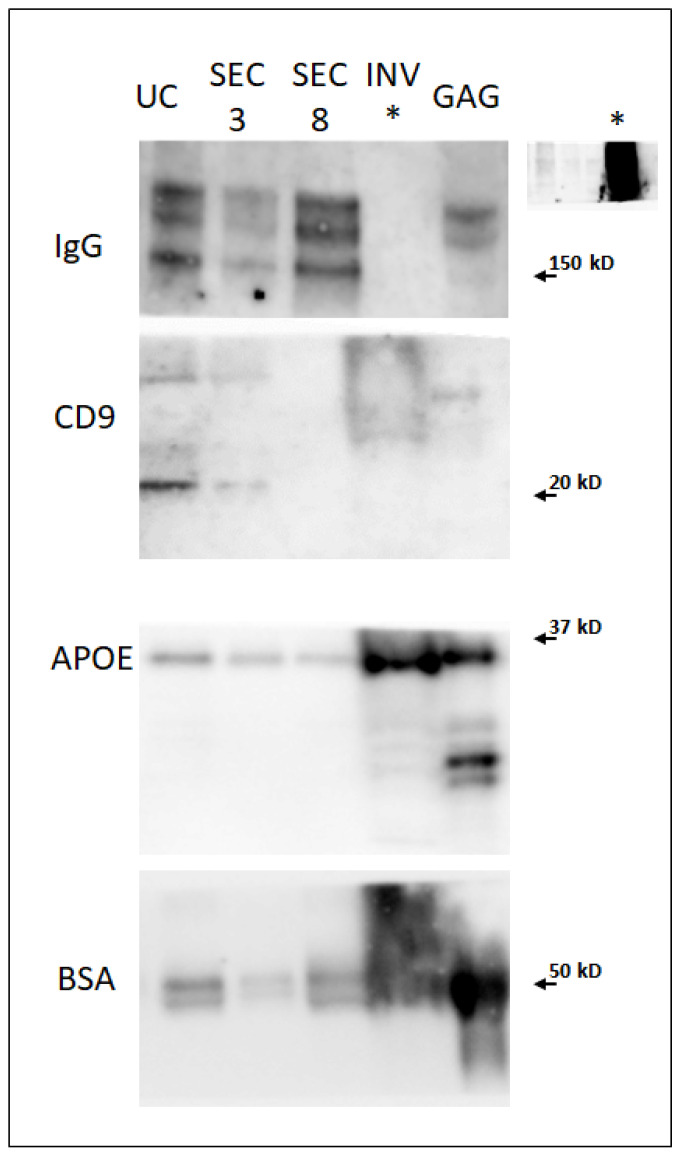
Western blot analysis of human EV-enriched serum preparations by using different procedures (UC, ultracentrifugation, SEC3, fractions 2–4 from SEC, SEC8 fractions 7–9 from SEC, INV, Total isolation solution from Invitrogen, Invitrogen, Thermo Fisher Scientific, Waltham, MA, USA, GAG, Exo-GAG precipitation solution from Nasabiotech, A Coruña, Spain). * In the case of the sample INV, it was not possible to assay the sample against IgG, due to overload of protein, as shown in the small picture of the gel at the right corner.

The observation of the samples by electron cryo-microscopy showed double membrane-bounded vesicles in the preparations obtained by all methods. However, there was also other non-vesicular electron dense material in the preparations, particularly in preparations obtained following INV and GAG techniques (Supplemental Figure S1). Western blot analysis detected the presence of the *bona-fide* EV associated protein CD9 only in UC and SEC3 (fractions 2–4 of the SEC, corresponding to vesicular fractions) but not in the rest of techniques (Figure 2). The apolipoprotein APOE was detected in all preparations, while proteins not associated to vesicles, such IgGs and BSA, were detected in all the samples although at different levels: their presence were more abundant in the samples precipitated with INV and GAG, and less profuse in the fraction SEC3 (Figure 2). The sample INV saturated the image for IgG (insert in Figure 1). NTA analysis revealed that UC is the technique that shows a smaller number of particles, and INV the highest, almost 100 times more than UC (Supplemental Figure S2).

### 2.2. Proteomic Profile of EVs Enriched by Ultracentrifugation

**Figure 3:**
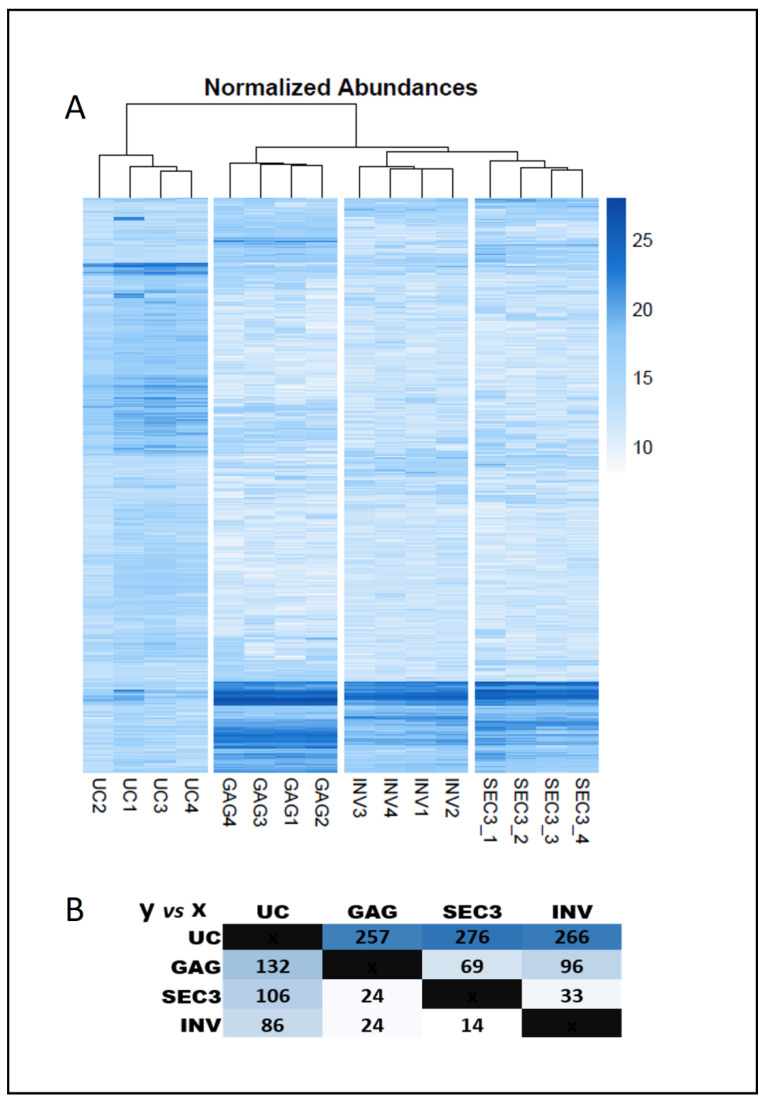
Differences in the proteomic profile enrichment achieved by each technique. (**A**) Unsupervised heatmap of normalized intensity values for each technique and identified protein. The color intensity correlates with the value of normalized abundance. (**B**) Proteins significatively enriched for each technique (sense of the comparison y axe vs. x axe) according to ANOVA and Tukey’s post hoc analysis (significance is considered for *p* value < 0.05, *n* = 4). The color intensity correlates with the number of molecules enriched.

The proteomic profile of the samples obtained by ultracentrifugation is the most differentiated among all the preparations. As observed in Figure 3A, the four UC samples constituted a separated branch in the clustering heatmap. More importantly, among the 578 proteins identified with at least two unique peptides for all preparations, 280 proteins are differentially represented in UC samples referred to the other 3 techniques, including 210 enriched, and 70 underrepresented, according to ANOVA and Tukey’s *post-hoc* analysis (total number of identified proteins, details of the identification, and ANOVA analysis is presented in Supplementary Table S1). A curated selection of the GO Cell Component analysis performed for those two groups of proteins are presented in Table 1. The proteins enriched in the UC preparations with respect to the other techniques are associated with the ribosome, the mitochondria, proteins related to focal adhesion, in addition to proteins associated to extracellular vesicles. About the proteins underrepresented in the UC preparations versus the rest of techniques, they include proteins associated with lipoproteins, immunoglobulins, platelet particles, and extracellular vesicles as well.

Figure 3B illustrates the number of proteins enriched in UC preparations versus each other technique, and it can be observed that in all the cases, UC had more proteins enriched than underrepresented, suggesting that UC isolated a larger number of proteins than other techniques. Conversely, the intensity heatmap of Figure 3A showed that the rest of techniques enriched certain proteins with larger abundance, in terms of signal intensity (darker blue color). Remarkably, UC technique seems to avoid the capture of some abundant serum proteins such immunoglobulins or lipoproteins.

### 2.3. Profile of Proteins Captured with a Precipitation Kit with Affinity for Glycosylated Groups

The GAG methodology enriches preparations that are also different from the other technologies, as presented in the dendrogram of Figure 3A. The preparations obtained by GAG are significantly enriched in a higher number of proteins than INV and SEC3 (Figure 3B). There are 45 proteins enriched with respect to the other techniques, and three underrepresented versus the other all techniques. The GO Cell Component analysis for those 45 proteins is presented in Table 2 showing that those proteins are related to circulating vesicles, as well as platelet granules and the membrane attack complex. The proteins more significantly enriched are a protein of the complement system, C1 protease inhibitor, prothrombin, and kininogen1, a protein related to blood coagulation. Regarding the underrepresented proteins, these are just three proteins named CAMP, an antibacterial peptide; PC, a pyruvate carboxylase; and RL18, a ribosomal protein.

**Figure 4:**
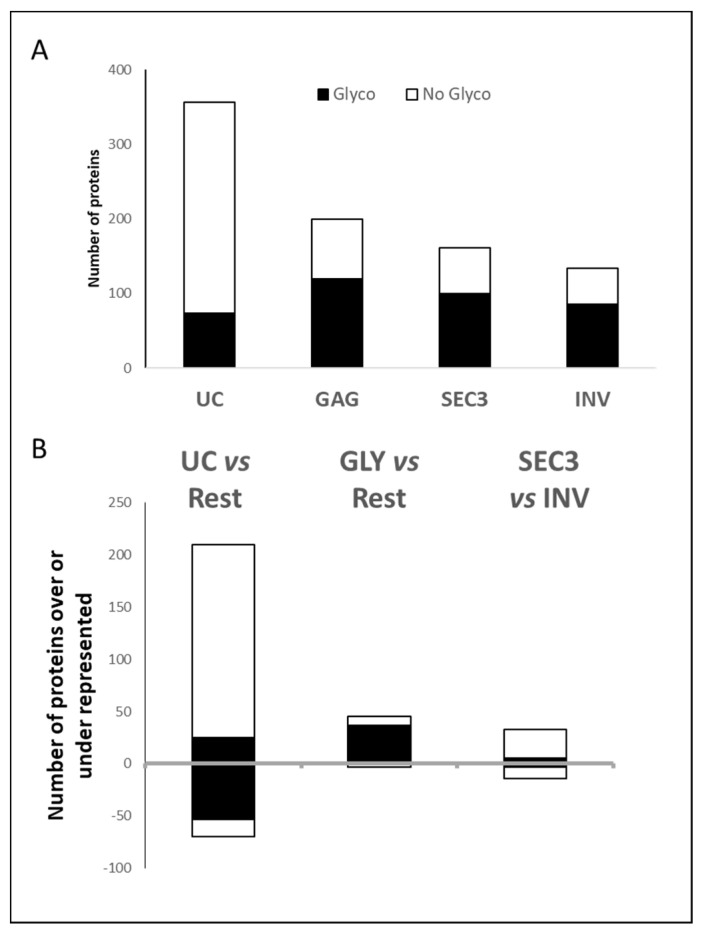
(**A**) The upper graph shows the number of proteins annotated in UNIPROT as harboring glycosylation sites (in black) within the total number of proteins detected for each technique (at least two peptides should be detected in 3 biological replicates). (**B**) The graph shows the number of proteins with glycosylated sites within the number of enriched or underrepresented proteins for each technique compared with the rest or between SEC3 and INV.

As the GAG isolation method is based upon affinity for glycosylated proteins, we have looked if the proteins enriched with GAG are more prone to harbor potential glycosylation sites (annotations obtained from UNIPROT, see Supplemental Table S1) when compared with the overall analysis and the proteins enriched with each technique. Among the 579 identified proteins, 556 are annotated in the database UNIPROT, and 173 are annotated as potentially susceptible of N- and O- glycosylation modifications, which is ~30% of proteins. For each technique, there are ~60% of proteins harboring glycosylation sites, except for UC where the percentage is just 20% (Figure 4). Just 10% of the proteins enriched in UC, in comparison with the rest of the techniques, harbor predicted N- and O-glycosylation sites. Among the underrepresented proteins, 75% of the proteins are potential targets of glycosylation. Regarding GAG, the percentage of potentially glycosylated proteins among the enriched proteins with this technique is 82%, confirming the affinity of this technique for glycosylated groups. With respect to SEC3 and INV preparations, they have a similar percentage of potentially glycosylated proteins in the proteins enriched for one technique with respect to the other, ~15%, which means that most of the glycosylated proteins detected in the preparations from these techniques are in similar abundance.

### 2.4. Differences between the Preparations Performed with Size-Exclusion-Based Method with Respect to the Polymer-Based Precipitation Method

As shown in Figure 3A, the preparations obtained by SEC3 and INV are the ones more similar, and they have less enriched proteins in total than the other two techniques. For that reason, the GO cell component analysis of Table 3 has been performed with the proteins enriched and underrepresented in SEC3 respect to INV. The result shows that SEC3 preparations were enriched in lipoproteins, while INV captures more elements from ribosomes and platelet granules.

In the case of size exclusion, it is also interesting to compare the proteins enriched in the SEC3 versus fractions SEC8 (the 7th to 9th collected fractions). Among all detected proteins, 32 were enriched in SEC3 with a fold change higher than 10 times, and a significant *p* value under 0.05. In addition to extracellular vesicles, lipoproteins, platelet granules, and IgA complex are other terms enriched in the vesicular fraction of the preparations obtained by SEC3 (see Table 4). Only five proteins were enriched in SEC8 with respect to SEC3, including CXCL7 (platelet basic protein) APOH, COX5B, Ig kappa chain V-III, and SCAM3, a secretory carrier-associated membrane protein.

## 3. Discussion

This study shows that commonly used EV enrichment techniques yield a very distinct protein profile. Although all the techniques isolate proteins associated with EVs, the GO Cell Component analysis indicates important differences in the proteins isolated. The preparations obtained though UC are enriched in a larger number of proteins when compared to other techniques, and the Cell Component analysis includes proteins associated to mitochondria and ribosomes, while lipoproteins and immunoglobulins are less prone to capture by this technique, as well as glycosylated proteins. It is important to mention that UC has been employed to differentiate the proteomic profile of patients of different liver diseases [17].

**Figure 5:**
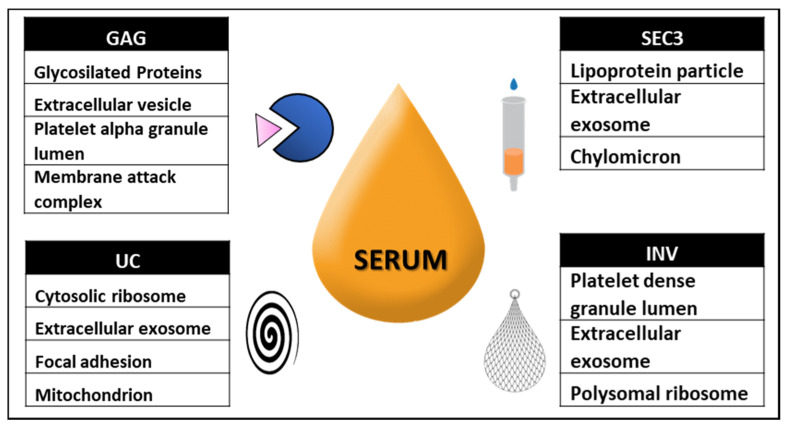
Summary of the cell component analysis associated with the proteins overrepresented in the preparations obtained with different isolation techniques.

On the other hand, GAG had a strong affinity for glycosylated proteins, as expected by their composition, and together with INV, the other precipitation method, showed enrichment in proteins associated with granules from platelets. As previously reported, the fractions associated with vesicles obtained from size exclusion usually carry over more proteins associated with lipoproteins when compared to other techniques (Figure 5).

In comparison with previous studies of proteomic profiles obtained from serum vesicles, we had observed several proteins shared with our study (Supplementary Table S2). In Table 5, we offer the number of common proteins between our studies and the Top100 proteins registered in the Vesiclepedia database (http://microvesicles.org/ accessed on 27 April 2021). Within that list, there are four proteins in common for all the techniques, named C3, a protein from the complement complex, FN1 (fibronectin), A2M (alpha-2-macroglobulin), and ACTB. Interestingly, UC is the methodology with a larger intersection with this database, perhaps because UC is the most popular method to isolate EVs [18]. To mention some of the proteins found in UC and the Top100 list, we found HSP90A, RAB7A, or GRP78, being the last one normally listed as a reporter of contamination from reticulum [19].

However, there is a larger overlap between the other techniques and the list of proteins described in [20]. That study lists more than 300 proteins detected by proteomics applied to SEC fractions of 500 µL of serum. In our case, we have detected one-third of those from 80 µL, and interestingly, employing both SEC and precipitation methods. In addition, through these three techniques, we were able to detect the presence of CDL5 and LGALS3BP, while UC did not detect them. These two proteins have been recognized as a hallmark of blood vesicles by [21], as they are not listed as serum proteins [20]. Their presence is very consistent in the proteomic analysis of blood circulating EVs, even in the very refined purification of plasma EVs combining SEC and density cushion [15]. The proteomics analysis performed in this last study identified many EV proteomic markers, such CD9, CD81, or CD63, that rarely can be detected by proteomics from blood-derived EVs. This was achieved thanks to the combination of SEC and cushion techniques and employing 6–12 mL of plasma. In Table 5, we show how many of our identified proteins match with both studies [15,20], and again UC is the study with less common proteins.

Moreover, note the presence in the UC preparations of some proteins listed both in the Top100 and the consensus list of [15,20], such as clathrin heavy chain 1 (CLTC), fatty acid synthase (FASN), and myosin-9 (MYH9), that in our case did not appear in the SEC preparation. This suggests that each technique can capture a different population of vesicles, and therefore it will bias for a specific plethora of markers. The application of UC to serum samples has been employed to find biomarkers of cholangiocarcinoma [22] and alcoholic and nonalcoholic fatty liver diseases, where significant differences in protein expression were found in apolipoproteins, immunoglobulins, and other previously reported markers of liver disease [17].

Nevertheless, the results showed the presence of serum proteins that are co-purify in the preparations. This phenomenon has been described in other studies [20], but as we have started from a lower amount of serum, the importance of contaminant proteins is more relevant. For that reason, we should emphasize that the proteomic profile does not just correspond to the vesicles but include several carry-over proteins. However, each methodology had specific affinity for different types of proteins and Figure 5 summarizes the result of such differences. In addition, any of the techniques employed was specific for a type of EVs, and accordingly, electron cryo-microscopy images showed vesicles of different sizes, including EVs larger than 100 nm, despite of the previous steps of centrifugation at 10,000× *g*. To finalize, we should remark that the present work has compared relatively simple, commonly available purification techniques, but it is likely that the field will evolve to more specialized purification techniques. We have already mentioned the purification combining SEC with density cushion [15], and it has been reported that flow field fractionation has an enormous potential to distinguish different vesicle populations [23,24]. In this context, it is relevant to bring the recent work applying fluorescence activated cell sorting and subsequent proteomics analysis on EVs from complex biofluids such tears and cerebrospinal fluid, recently published in the field of multiple sclerosis biomarker research [25].

In conclusion, our study shows that it is possible to identify a good number of vesicle-associated proteins through proteomics analysis starting with less than 100 µL of serum, which could be very interesting for both clinical applications and laboratory animal studies. However, the technique employed to isolate vesicles will determine the proteomic profile observed in a higher degree, and therefore will bias the results in one or another direction.

## 4. Materials and Methods

### 4.1. Obtention of Human Serum Samples

Serum was obtained from healthy donors from the BASQUE BIOBANK repository in frozen aliquots (CEIC-PI2016037). Different aliquots from individuals were generated, each of them with 350 µL of serum plus 150 µL of PBS. The diluted samples were cleared by centrifugation at 2000× *g* per 5′, and then at 10,000× *g* per 20′ in a top table Optima ultra-centrifuge with a TL-120 rotor (Beckman Coulter, Pasadena, CA, USA) to remove larger vesicles.

### 4.2. Enrichment of Extracellular Vesicles

To produce the sample named UC, an aliquot of 100 µL of the cleared serum (approximately 80 µL of original serum) prepared as described above was subsequently diluted with 400 µL of PBS and ultracentrifuged for 75 min at 100,000× *g* in a TL-120 rotor (Beckman coulter). The pellet was washed with 500 µL of PBS and centrifuged again. Finally, the pellet was resuspended in a final volume of 50 of PBS (Western blotting, NTA, or ME assay) or cell lysis buffer (CLB, composed of 7 M urea, 2 M thiourea and 4% CHAPS) for proteomics analysis.

An aliquot of 100 µL of cleared serum was loaded in a SEC column and fractionated by modifying an existing published protocol [26]. Briefly, Sepharose CL-2B (Sigma-Aldrich, San Luis, MI, USA) was packed in a poly-prep chromatography column (Bio-Rad, Hercules, CA, USA) with 2 mL of bed volume. The cleared serum was allowed to enter in the column, and eluted with 2 mL of PBS, collecting fractions of 200 µL each. Fractions 2 to 4 were pooled (named SEC3), and fractions 7 to 9 were pooled and named SEC8. For Western blotting, electron microscopy, and NTA analysis, the fractions were ultracentrifuged at 100,000× *g* for 1 h, and pellet resuspended in 50 µL of PBS. For proteomics analysis, the proteins in the pooled fractions were extracted by adding 3 mL of ice-cold acetone, kept at −20 °C overnight, and finally centrifuged at 3000× *g* for 30′. The pellet was resuspended with 50 µL of CLB.

The purification with the Total Exosome Purification kit (Invitrogen, Thermo Fisher Scientific, Waltham, MA, USA) was performed according to the manufacturer’s indications. To each aliquot of 100 µL of cleared serum was added 50 µL of PBS and 30 µL of precipitation reagent, mixed thoroughly, and incubated for 10′. Afterwards, the solution was centrifuged for 5′ at 10,000× *g* and the pellet was resuspended with 50 µL of PBS (Western blot, NTA, or ME assay) or CLB (proteomic analysis).

The purification with Exo-GAG precipitation solution (Nasabiotech, A Coruña, Spain), named GAG, was performed according to the manufacturer’s indication. To each aliquot of 100 µL of cleared solution, 200 µL of precipitation reagent was added, mix it thoroughly, and incubated on ice for 5′. Afterwards, the sample was centrifuged at 15,000× *g* during 15 min and the pellet was resuspended with 50 µL of PBS (Western blotting, NTA, or ME assay) or CLB (proteomic analysis).

### 4.3. EVs Characterization by Electron Microscopy and Nanoparticle-Tracking Analysis

For cryo-electron microscopy, EV preparations were directly adsorbed onto glow-discharged holey carbon grids (QUANTIFOIL, Jena, Germany). Grids were blotted at 95% humidity and rapidly plunged into liquid ethane with the aid of VITROBOT (Maastricht Instruments BV, Maastricht, The Netherlands). Vitrified samples were imaged at liquid nitrogen temperature using a JEM-2200FS/CR transmission electron microscope (JEOL, Akishima, Japan) equipped with a field emission gun and operated at an acceleration voltage of 200 kV.

Size distribution within EV preparations was analyzed using the nanoparticle-tracking analysis (NTA), by measuring the rate of Brownian motion in a NanoSight LM10 system (Malvern Panalytical, Malvern, UK). The system was equipped with a fast video-capture and particle-tracking software. NTA post-acquisition settings were the same for all samples. Each video was analyzed to give the mean, mode, and median vesicle size, as well as an estimate of the concentration.

### 4.4. Western Blot Analysis

PBS-resuspended EVs were mixed with 4×NuPAGE LDS Sample Buffer (Thermo Fisher Scientific, Waltham, MA, USA). The samples were incubated for 5 min at 37 °C, 65 °C, and 95 °C, and separated on 4–12% precast gels (from Thermo Fisher Scientific, Waltham, MA, USA), and transferred into PVDF membranes with the iBLOT2 system (Thermo Fisher Scientific, Waltham, MA, USA). Antibodies employed were mouse monoclonal antibody against CD9 #209302 (R&D), APOE #4E4 (Thermo Fisher Scientific, Waltham, MA, USA) and sheep polyclonal antibody against human serum albumin (Abcam). All the primary antibodies were diluted 1:1000.

### 4.5. Proteomics Analysis

In-solution protein digestion was performed following the FASP protocol described by Wisniewski et al. with minor variations [27], using Amicon Ultra 30K devices (Merck-Millipore, Burlington, MA, USA). Proteins were quantified with Bradford assays (Bio-Rad) prior to the addition of trypsin, and then incubated overnight at 37 °C. The resulting peptides were dried and resuspended in 0.1% formic acid and sonicated for 5 min prior to analysis.

Samples were analyzed in a novel hybrid trapped ion mobility spectrometry—quadrupole time-of-flight mass spectrometer (timsTOF Pro with PASEF, Bruker Daltonics, Bruker, Billerica, MA, USA) coupled online to a nanoElute liquid chromatograph (Bruker). Sample (200 ng) was directly loaded in a 15 cm Bruker nanoElute FIFTEEN C18 analytical column (Bruker, Billerica, MA, USA) and resolved at 400 nL/min with a 30 min gradient.

Raw MS files were analyzed using MaxQuant v. 1.6.12.0. software. Proteins were identified matching to a human (Uniprot/Swissprot *Homo sapiens,* accessed on 16 April 2020) with a maximum of 2 missed cleavages and with precursor and fragment tolerances of 20 ppm and 0.05 Da. Only proteins identified with at least one peptide at FDR < 1% were considered for further analysis.

### 4.6. Cell Component Enrichment Analysis and Information on Glycosylation Sites

The list of proteins was loaded into g:Profiler [28] database and enrichment analysis was performed for GO Cell Components. The list of significant cell components was manually curated according to the strength, redundancy, and importance of the terms. The annotation of the presence of potential glycosylation sites for each protein was downloaded from UNIPROT (https://www.uniprot.org/ accessed on 18 June 2021).

## Figures and Tables

**Table 1:** GO Cell Component analysis of UC differentially enriched proteins vs. rest of techniques. Red color denotes enrichment in UC preparations, and blue color that the group is underrepresented.

#Term ID	Term Description	Term Size	Intersected	*p* Adjusted
GO:0022626	cytosolic ribosome	104	46	3.05 × 10^−59^
GO:0070062	extracellular exosome	2177	100	5.52 × 10^−34^
GO:0005925	focal adhesion	384	38	1.48 × 10^−21^
GO:0005739	mitochondrion	1380	66	5.04 × 10^−21^
GO:0015934	large ribosomal subunit	113	24	5.84 × 10^−21^
GO:0031966	mitochondrial membrane	573	44	7.98 × 10^−21^
GO:0072562	blood microparticle	138	44	2.97 × 10^−79^
GO:0070062	extracellular exosome	2177	51	4.92 × 10^−34^
GO:0062023	collagen-containing extracellular matrix	381	23	1.84 × 10^−20^
GO:0034358	plasma lipoprotein particle	23	7	2.93 × 10^−10^
GO:0031093	platelet alpha granule lumen	66	9	3.79 × 10^−10^
GO:0005579	membrane attack complex	6	5	7.24 × 10^−10^
GO:0042571	immunoglobulin complex, circulating	75	9	1.26 × 10^−9^
GO:0034361	very-low-density lipoprotein particle	15	6	1.91 × 10^−9^
GO:0034385	triglyceride-rich plasma lipoprotein particle	15	6	1.91 × 10^−9^

**Table 2:** GO Cell Component analysis of proteins enriched using GAG vs. preparations obtained with the rest of techniques. Red color denotes enrichment in GAG preparations.

#Term ID	Term Description	Term Size	Intersected	*p* Adjusted
GO:0072562	blood microparticle	138	21	3.78 × 10^−31^
GO:1903561	extracellular vesicle	2262	32	1.37 × 10^−17^
GO:0031093	platelet alpha granule lumen	66	9	1.15 × 10^−11^
GO:0005579	membrane attack complex	6	4	1.15 × 10^−7^

**Table 3:** GO Cell Component analysis of the proteins enriched or underrepresented in SEC3 vs. INV. Red color denotes enrichment in SEC3 preparations, and blue color that the group is underrepresented in SEC3 vs. INV.

#Term ID	Term Description	Term Size	Intersected	*p* Adjusted
GO:0072562	blood microparticle	138	20	1.13 × 10^−^^32^
GO:1990777	lipoprotein particle	23	8	5.31 × 10^−^^15^
GO:0070062	extracellular exosome	2177	25	8.04 × 10^−^^15^
GO:0042627	chylomicron	10	5	8.13 × 10^−^^10^
GO:0031089	platelet dense granule lumen	14	2	0.00270
GO:0070062	extracellular exosome	2177	7	0.00418
GO:0042788	polysomal ribosome	31	2	0.013765

**Table 4:** GO Cell Component analysis of molecules enriched in SEC3 vs. SEC8 preparations. Red color denotes enrichment in SEC3 preparations.

#Term ID	Term Description	Term Size	Intersected	*p* Adjusted
GO:0072562	blood microparticle	138	22	1.47 × 10^−^^38^
GO:0070062	extracellular exosome	2177	21	1.16 × 10^−^^10^
GO:0071746	IgA immunoglobulin complex, circulating	5	3	1.03 × 10^−^^5^
GO:1990777	lipoprotein particle	23	4	1.39 × 10^−^^5^
GO:0031093	platelet alpha granule lumen	66	5	2.01 × 10^−^^5^

**Table 5:** Overlap between our study and the Top-100 proteins described in Vesiclepedia, and with previous published proteomics analysis of blood samples. Intensity of the color correlates with the numerical value within columns. To consider a protein identified, at least two peptides should be detected in 3 biological replicates.

	Identified 3 Replicates	Top 100 Vesiclepedia	Ref [16]	Ref [11,16]
UC	342	28	105	49
GAG	194	16	114	85
SEC3	157	8	115	86
INV	131	6	108	76

## Data Availability

The mass spectrometry proteomics data have been deposited to the ProteomeXchange Consortium via the PRIDE partner repository. Data are available via ProteomeXchange with identifier PXD029015.

## Supplementary Materials

The following are available online at www.mdpi.com/article/10.3390/ijms222011144/s1.
