# Supplementary material for: Human Serum Extracellular Vesicle Proteomic Profile Depends on the Enrichment Method Employed
Source: Int J Mol Sci. 2021 Oct 15;22(20):11144. doi: 10.3390/ijms222011144 (PMC8540106; doi:10.3390/ijms222011144)

Supplementary Figure S1. Cryo-electron microscopy of serum samples obtained by the different techniques.

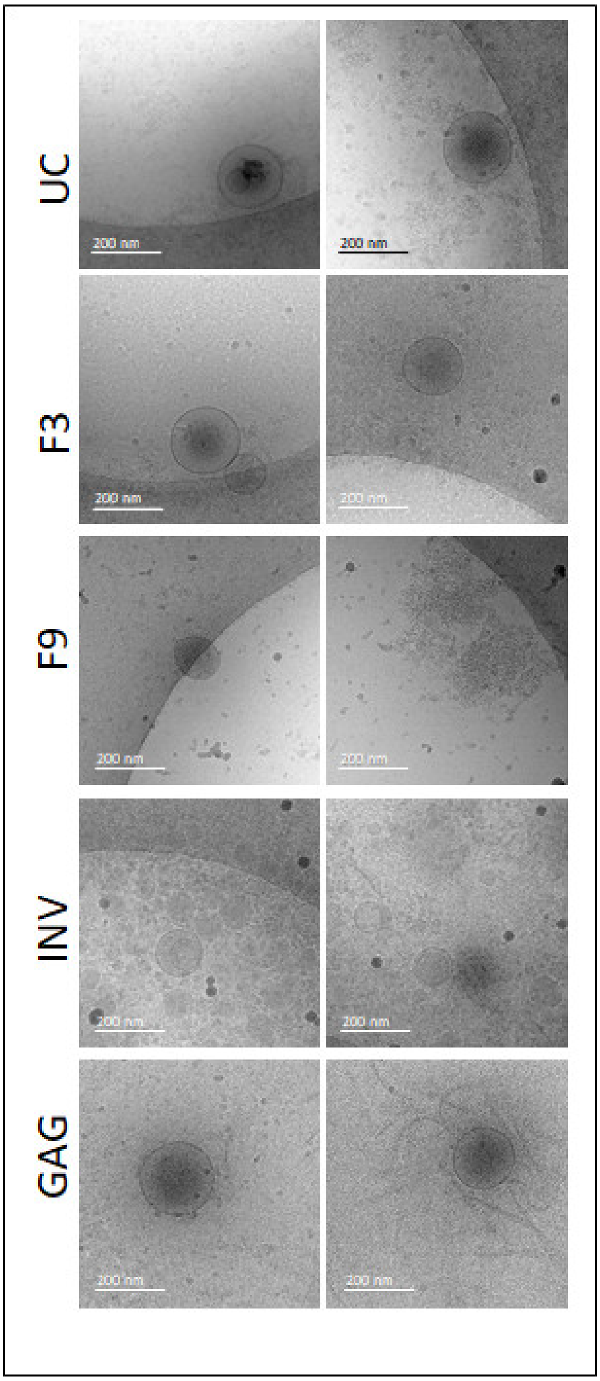

Supplementary Figure S2. NTA profiling of the preparations obtained by different techniques, and the concentration obtained.

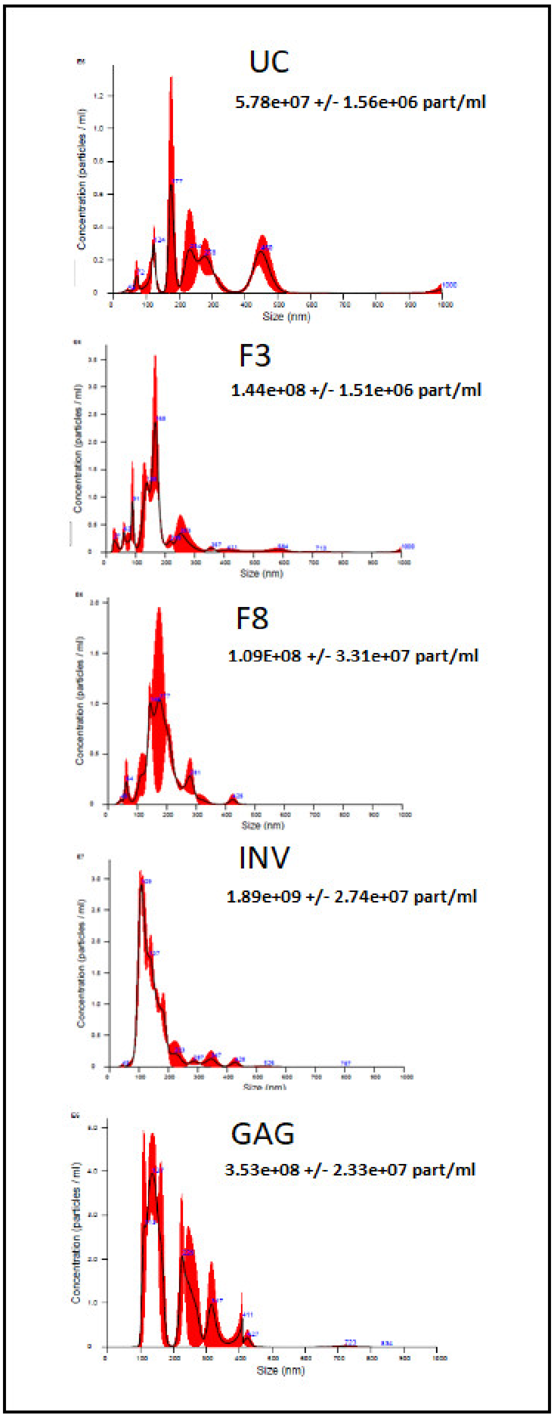

Supplement: Supplementary file 1 [file ijms-22-11144-s001.zip › Supplemental Figures IJMS_v1.pdf]
